# Plasma D-dimer is not useful in the prediction of deep vein thrombosis after total knee arthroplasty in patients using rivaroxaban for thromboprophylaxis

**DOI:** 10.1186/s13018-018-0883-1

**Published:** 2018-07-11

**Authors:** Cheng-Ta Wu, Bradley Chen, Jun-Wen Wang, Shih-Hsiang Yen, Chung-Cheng Huang

**Affiliations:** 1 Department of Orthopaedic Surgery, Kaohsiung Chang Gung Memorial Hospital, 123, Ta Pei Road, Niao Sung District, Kaohsiung, Taiwan, Republic of China; 2 Institute of Public Health, National Yangming University, Taipei, Taiwan, Republic of China; 3 College of Medicine, Chang Gung University, 123, Ta Pei Road, Niao Sung District, Kaohsiun0067, Taiwan, Republic of China; 4 Department of Radiology, Kaohsiung Chang Gung Memorial Hospital, Kaohsiung, Taiwan, Republic of China

**Keywords:** D-dimer, Total knee arthroplasty, Venous thromboembolism, Thromboprophylaxis, Rivaroxaban, Deep vein thrombosis

## Abstract

**Background:**

Venous thromboembolism (VTE) is a serious complication following total joint replacement. The use of rivaroxaban, a highly selective and direct factor Xa inhibitor, has been used widely as a safe and efficacious way to prevent VTE after total joint replacements. However, little is known about the diagnostic efficacy of plasma D-dimer test on deep vein thrombosis (DVT) in patients using rivaroxaban for thromboprophylaxis. The study is aimed to investigate the trend and the diagnostic efficacy of D-dimer test on DVT in patients with primary total knee arthroplasty (TKA) using rivaroxaban for thromboprophylaxis.

**Methods:**

Two hundred TKA patients using rivaroxaban postoperatively as chemical prophylaxis were reviewed. D-dimer levels were checked at 4 h after the surgery and on postoperative days 1 and 4. Venography was used to document the presence of DVT. The Mann-Whitney *U* test was used to detect the differences in the D-dimer levels at different time points in patients with and without DVT, followed by Bonferroni corrections for *p* values. Receiver operating characteristics (ROC) curves were constructed to determine the best cutoff values of the D-dimer test at each time point after the surgery.

**Results:**

Twenty-nine of the 200 patients were found to have deep vein thrombosis by venography, resulting in an incidence of 14.5%. All patients with DVTs occurred in the distal calf veins, and only one patient was symptomatic. We found significant differences in D-dimer concentration between patients with and without DVT at postoperative day 4. The best cutoff value determined by receiver operating characteristics analysis was 3.8 mg/L at postoperative day 4, with an AUC equal to 63.5%, and a sensitivity, specificity, PPV, and NPV of 58.6, 76, 29.3, and 91.5%, respectively.

**Conclusions:**

Rivaroxaban was effective on reducing DVT in patients undergoing TKA. Because all the DVTs occurred in the leg veins, decreased thrombus volume and size might result in poor accuracy of plasma D-dimer test in prediction or diagnosis of postoperative DVT.

## Background

Venous thromboembolism (VTE) is a potentially serious complication following total joint replacement. The overall incidence of deep vein thrombosis (DVT) following total joint replacement in patients without thromboprophylaxis ranged around 40~70% in Western countries [1–3]. Similar prevalence has been reported in Asian populations [4–6]. While most of the patients were asymptomatic [7], propagation of the thrombi in the untreated patients could result in potential fatal outcome such as pulmonary embolism [8]. Early diagnosis of DVT is important in that timely pharmaceutical intervention with anticoagulants reduces the morbidity and mortality from VTE [9, 10]. However, accurate diagnosis of DVT remains challenging to clinical physician since the patients’ symptoms and signs are unreliable. Venography and compressive ultrasonography are the two most often used modalities to diagnose the DVT. Nevertheless, certain limitations hamper their widespread application as screening tools. Despite of being considered the gold standard in the diagnosis of DVT in lower extremity [11], venography is a costly and invasive procedure carrying certain risk [3, 12]. On the other hand, the sensitivity of ultrasonography for distal and non-occlusive proximal DVT was reported to be less favorable and operator-dependent [12, 13].

As a plasma marker specific to endogenous fibrinolysis, D-dimer has been demonstrated to be sensitive and helpful in the diagnosis of DVT [6, 9, 14, 15]. D-dimer is the proteolytic end-product formed by the action of plasmin on cross-linked fibrin in the presence of calcium. Therefore, with only few exceptions, elevated D-dimer level is indicative of a process of fibrin formation and dissolution in ongoing thrombosis such as DVT [16]. It carries a neo-antigen that is different from the parent fibrinogen molecule. By means of detecting the neo-antigen with certain monoclonal antibodies, current commercially available assays are able to quantify the level of D-dimer in a simple and efficient way [15, 17]. In addition, measurement of D-dimer was reported to have the economic potential to spare the use of other expensive tests such as venography and ultrasonography [6]. Despite of its clinical and economic advantages, the diagnostic efficacy of D-dimer test remains controversial in patients undergoing total knee arthroplasty (TKA). Previous studies have shown that the test had a high negative predictive value to exclude the presence of DVT [10, 18]. However, high false-positive rate after major orthopedic surgery hampered its usefulness [9]. Several factors other than DVT such as surgical trauma itself and the use of pneumatic tourniquet could elevate D-dimer level [15, 16, 19]. This resulted in a questionable interpretation of D-dimer test for early detection of DVT after TKA. Some studies have shown a correlation of elevated D-dimer test on postoperative days 4 or 7 after TKA with the occurrence of DVT [6, 9, 14]. However, most of the studies did not employ chemical thromboprophylaxis after the surgery. Niimi et al. reported the accuracy of D-dimer test to be less favorable in predicting DVT if fondaparinux, an injectable form of factor Xa inhibitor, was administered after TKA [20].

Rivaroxaban, one of the first licensed oral factor Xa inhibitors, has recently been used widely as a practical and efficacious way to prevent VTE after total joint replacements. Its influence for plasma D-dimer measurement on the diagnosis of postoperative DVT has not been investigated. The aim of this retrospective study is to investigate the trend and the diagnostic efficacy of D-dimer test on DVT in TKA patients using rivaroxaban for thromboprophylaxis.

## Methods

### Patients

Between August 2012 and April 2014, 294 eligible patients scheduled to undergo primary TKA for advanced osteoarthritis and postoperative venography for DVT screening were reviewed in retrospect. Patients were included in the analysis if they were (1) equal or older than 18 years of age, (2) undergoing unilateral primary TKA, and (3) documented with or without lower limb DVT after the surgery by ascending venography. Patients were excluded if they had (1) coagulopathy (such as hemophilia or thrombocytopenia); (2) significant liver disease; (3) severe renal impairment (creatinine clearance < 30 ml/min); (4) concomitant use of protease inhibitors of human immunodeficiency virus, or fibrinolytic agents that was contraindicated to the use of rivaroxaban; (5) prior surgery on the affected knee; (6) a history of thromboembolic disease requiring life-long anticoagulant therapy or anti-platelet drugs that could not be stopped before operation, and (7) no venographic report due to technical failure or refusal to receive the exam. As a result, 200 patients who fulfilled the criteria mentioned above were enrolled in the analysis. The study was conducted with a waiver of patient consent and approved by the Institution Review Board of our hospital.

### Perioperative management and DVT prophylaxis

The demographics of the patients, including age, gender, body mass index (BMI), American Society of Anesthesiologists (ASA) grade, and types of anesthesia, were recorded. All patients completed routine preoperative work-up, including complete blood count, chemistry profiles, and coagulation profiles. Physical examination and preoperative D-dimer test precluded DVT before the surgery. All operations were performed under general or spinal anesthesia with a pneumatic tourniquet inflated to a pressure of 300 mmHg before the incision and released at the end of surgery after skin closure. The components of the TKA prostheses were all fixed with cemented technique. All patients received 10 mg of oral rivaroxaban (Xarelto, Bayer Shering Pharma AG, Wuppertal, Germany) once daily from postoperative day (POD) 1 to POD 14 according to the ACCP guideline for VTE prophylaxis in TKA patients [21]. No other modalities for VTE prophylaxis such as pneumatic compressive devices were used. Postoperative rehabilitation commenced from continuous passive motion of the knee after returning to the ward, followed by physical therapy for muscle strengthening and partial weight bearing ambulation with walker on the next day of the surgery. The hemovac was routinely removed on POD 2. The patients were allowed for hospital discharge if they were independent on ambulation with walker support and the operated knee joint reached a range of motion > 90°. All patients returned for follow-up at 2 weeks, 3 months, and 6 months after the surgery.

### Laboratory data and venography

Preoperative data, including hemoglobin (Hb) level, prothrombin time, activated partial thromboplastin time, D-dimer level, and platelet count were collected. The Hb level was followed on POD 1, 2, and 4. The D-dimer levels were measured at 4 h after the end of the surgery, on POD 1 and POD 4. Total Hb loss was calculated by subtracting the lowest Hb level after operation from the preoperative Hb level based on the assumption that blood volume was normalized on POD 4. Total blood loss was calculated according to the method of Nadler et al. [22], which used the maximum postoperative reduction in Hb level adjusted for weight and height of the patient and the following formula: Total blood loss = (Total blood volume × [change in Hb level/preoperative Hb level]) × 1000 + volume transfused.

The measurement of plasma D-dimer level was performed with the INNOVANCE ® D-Dimer (Siemens Healthcare Diagnostics Products GmbH, Marburg, Germany) immunoturbidimetric assay, which used a monoclonal antibody (8D3) to detect and quantify only cross-linked D-dimer fragments.

Bilateral ascending venography of the legs was carried out on the next day after the last dose of rivaroxaban, or earlier if symptomatic, using the Rabinov and Paulin technique [23]. A positive diagnosis of DVT required the demonstration of filling defect signs in contrast-filled veins or cutoff signs of one or several deep veins (indirect signs of DVT) [14]. Computed tomographic angiography of the chest was performed if pulmonary embolism was suspected. All the radiographic images were interpreted by an independent radiologist (CCH).

### Statistical analysis

Patients were divided into DVT and non-DVT groups based on the results of venography. Student’s *t* test was used for comparison of continuous variables between the two groups in the distribution of demographic and baseline data (including age, BMI, total blood loss, and preoperative laboratory data). The *χ*2 test or Fisher exact test was used when analyzing the differences of dichotomous variables between the two groups (including gender, ASA score ≥ 3, types of anesthesia, and numbers of patients with blood transfusion). The results were expressed as the mean ± standard deviation.

The Mann-Whitney *U* test was used to detect the differences in the D-dimer levels at different time points in patients with and without DVT, followed by Bonferroni corrections for *p* values. Receiver operating characteristics (ROC) curves were constructed to determine the best cutoff values of the D-dimer test at each time point after the surgery. The sensitivity, specificity, and positive and negative predictive values of the D-dimer levels were calculated using the standard method of proportions. All tests were two-sided, and *p* < 0.05 was considered significant. All statistical comparisons were made using the Statistical Package for Social Sciences (SPSS) (version 22; SPSS Inc., Chicago, Illinois).

## Results

### The incidence of DVT

DVT was identified by venography in 29 of 200 patients with primary TKA resulting in an incidence of 14.5%. All 29 patients with DVTs occurred in the distal calf veins, and no patients developed proximal DVTs detected by venography. The distribution of thrombosis of the leg veins of the 29 patients was described (Table 1).

**Table 1:** Distribution of muscular, combined, and major leg vein DVT

	No. of patients	Percentage
Proximal DVT	0	0%
Distal DVT	29	
Isolated muscular branches	9	31%
Major leg veins	14	48%
Combined muscular and major leg veins	6	21%

Muscular branches included gastroneumus and soleus muscular veins; major leg veins included anterior and posterior tibial and peroneal veins

There was only one patient had leg edema and calf tenderness 1 month after the operation. Ascending venography revealed thrombosis over the peroneal vein of the operated leg. No pulmonary embolism or VTE-related complication occurred within 15 days following the surgery. There were no significant differences between DVT (+) and DVT (−) groups in terms of sex, age, percentage of patients with ASA score ≥ 3, BMI, and types of anesthesia (Table 2).

**Table 2:** Comparison of demographic parameters of patients with and without DVT

	DVT		
Variables	Yes	No	*p* value
	(*N* = 29)	(*N* = 171)	
Male sex (F/M)	25/4	124/47	0.12
ASA score ≥ 3 [no./total no.(%)]	13/29(44.8%)	61/171(35.7%)	0.35
Age (years)	71.0 ± 6.9	69.0 ± 7.0	0.16
BMI (kg/m^2^)	26.5 ± 3.9	27.8 ± 3.5	0.07
Obesity (BMI ≥ 27, no.)	15	102	0.42
Pre-operative laboratory data			
Hemoglobin (g/dl)	12.6 ± 0.9	13.4 ± 1.1	< 0.01
Platelet count (10^9^cells/L)	249.2 ± 59.8	228.7 ± 55.1	0.27
PT INR	1.0 ± 0.04	1.0 ± 0.6	0.67
aPTT INR	1.0 ± 0.1	1.0 ± 0.1	0.28
Types of anesthesia (no.)			0.61
General anesthesia	25	139	
Spinal anesthesia	4	32	
Total blood loss (ml)	989.8 ± 300.3	1056.6 ± 335.7	0.32
Blood transfusion	0/29(0%)	8/171(4.7%)	0.61

All data are expressed as mean ± standard deviation

*ASA* American Society of Anesthesiologists, *BMI* Body mass index, *INR* International normalized ratio, *PT* Prothrombin time, *aPTT* Activated partial thromboplastin time

### D-dimer level and DVT

**Fig. 1:**
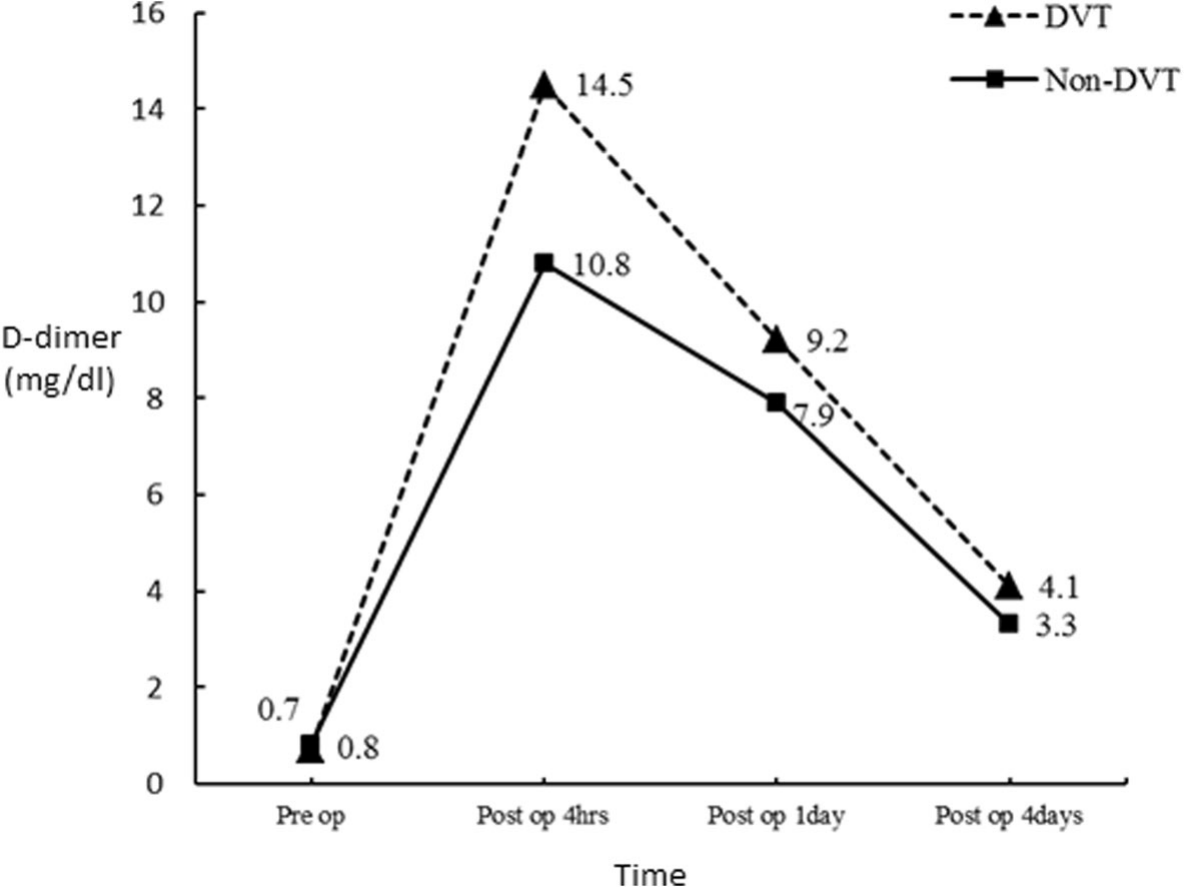
Longitudinal changes of the D-dimer levels after TKA. The D-dimer levels reached a peak value at 4 h after the surgery and decreased gradually on POD1 and POD4 in both DVT (+) and DVT (−) groups. The D-dimer levels were significantly higher in patients with DVT than in patients without DVT on POD4

There were no significant differences in the pre-operative D-dimer concentrations between patients with postoperative DVT and without DVT (median, 0.5 mg/L vs. 0.5 mg/L, *p* = 0.97). In both groups, the levels of D-dimer rose markedly at 4 h immediately after the surgery (*p* < 0.01) and decreased gradually on POD1 and POD4 (Fig. 1). The plasma concentrations of D-dimer were found significantly higher in DVT (+) group than in DVT (−) group on POD4 (median, 4.0 mg/L vs. 3.3 mg/L, *p* = 0.04). The correlations of D-dimer levels and the occurrence of venographic DVT were shown in Table 3.

**Table 3:** Correlations of D-dimer levels and DVT

	DVT		
Variables	Yes	No	*p* value^a^
	(*N* = 29)	(*N* = 171)	
Post-operative laboratory data:			
Pre-op D-dimer (mg/L)	0.5 (0.21–2.87)	0.5 (0.19–6.11)	0.97
Post-op 4 h D-dimer (mg/L)	9.2 (2.45–35.13)	8.5 (1.4–35)	0.56
Post-op day 1 D-dimer (mg/L)	8.2 (1.35–26.5)	5.6 (0.19–34.47)	0.26
Post-op day 4 D-dimer (mg/L)	4.0 (1.66–9.81)	3.3 (0.74–9.4)	0.04

The data are expressed as medians (min–max)

^a^The *p* values are expressed after Bonferroni corrections

### Diagnostic efficacy of D-dimer

**Fig. 2:**
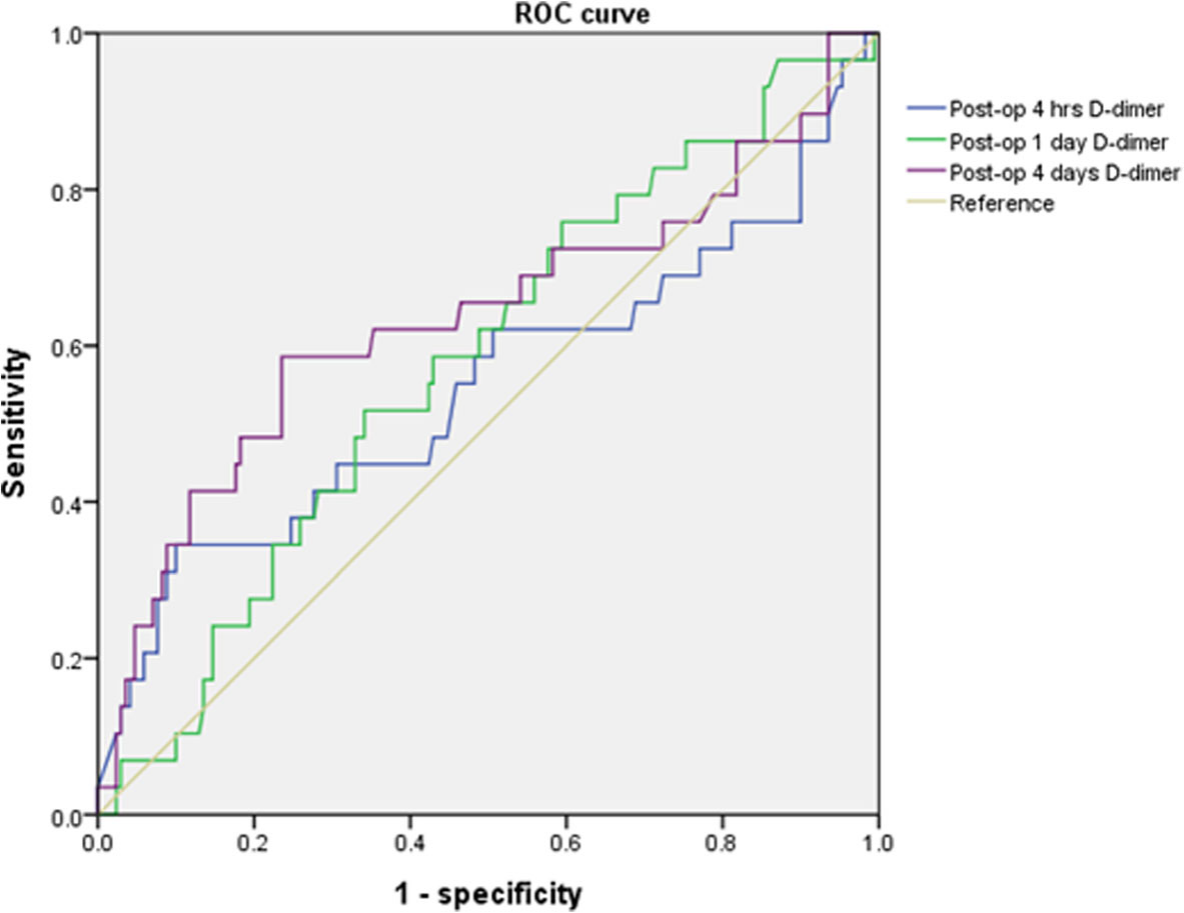
Receiver operating characteristics (ROC) curves for plasma D-dimer in the diagnosis of DVT. The AUCs of plasma D-dimer at 4 h after surgery, POD1, and POD4 were 0.547 (*p* = 0.42), 0.58 (*p* = 0.17), and 0.635 (*p* = 0.02), respectively. AUC, area under curve

Receiver operating characteristics (ROC) analyses of the D-dimer tests at each time point after the surgery were constructed (Fig. 2). The ROC curve obtained for various cutoff values of D-dimer levels at 4 h after the surgery determined a best cutoff value of 11.2 mg/L, yielding a sensitivity of 44.8%, specificity of 69.6%, PPV of 20%, and NPV of 88.1%. However, the area under the curve (AUC) was 54.7% and was statistically insignificant (*p* = 0.42). The best cutoff value at 1 day after the surgery was 8.2 mg/L, and the AUC was equal to 58% (*p* = 0.17). The best cutoff value at 4 days after the surgery was 3.8 mg/L, with an AUC equal to 63.5% (*p* = 0.02), and a sensitivity, specificity, PPV, and NPV of 58.6, 76, 29.3, and 91.5%, respectively (Table 4).

**Table 4:** Cutoff values, sensitivity, specificity, and predictive values of D-dimer

D-dimer test	Best cutoff value (mg/L)	Sensitivity (%)	Specificity (%)	Positive (%)	Negative (%)	AUC (%)	*p* value
				Predictive value			
Post-op 4 h	11.2	44.8	69.6	20.0	88.1	54.7	0.42
Post-op 1 day	8.2	51.7	64.9	20.0	88.8	58.0	0.17
Post-op 4 days	3.8	58.6	76.0	29.3	91.5	63.5	0.02

*AUC* area under curve

## Discussion

In this study, we have found a high peak value of D-dimer concentration at 4 h after surgery and decreased gradually on POD1 and POD4 in both DVT (+) and DVT (−) groups. Patients undergoing TKA sustained a higher incidence of postoperative DVT as compared with total hip arthroplasty (THA) owing to the use of tourniquet [24–26]. Prior venographic studies reported that the rates of DVT ranged from 30 to 57% for THA and from 40 to 84% for TKA in the absence of thromboprophylaxis [4, 5, 27]. A meta-analysis study also demonstrated that TKA patients managed with a tourniquet had higher risks of thromboembolic complications [28]. Inadequate exsanguination of the blood in the limb, stasis, and ischemia contribute to thrombosis formation [29]. Circulatory indices of thrombosis such as D-dimer values significantly increase immediately following deflation of the tourniquet after TKA [30]. It may decline 1 to 3 days postoperatively [31].

The usefulness of plasma D-dimer measurement in the prediction or diagnosis of postoperative DVT in asymptomatic patients after major orthopedic surgeries has been a controversial issue, as well as the cutoff value of the D-dimer level. Shiota et al. reported that over 10 mg/L of D-dimer concentration on POD7 indicated occurrence of DVT after lower limb arthroplasty [14]. Jiang et al. concluded that plasma D-dimer level was a useful screening test to exclude DVT after orthopedic surgery [32]. However, other authors reported the contradictory results [2, 33, 34]. The variability of timing of D-dimer measurement, selection of the patients, the diagnostic modalities of DVT, and with or without chemical anticoagulants postoperatively may result in the different conclusions.

The second finding of our study was that the D-dimer values were higher in DVT (+) group than DVT (−) group at 4 days after the surgery (*p* = 0.04). Similar finding was reported by Niimi et al. who found that D-dimer values were higher in patients with DVT than in patients without DVT on POD 7 (*p* < 0.01) when fondaparinux was used after THA and TKA [20]. They reported that the cutoff value of D-dimer at POD7 in patients treated with fondaparinux was 6.04 μg/ml, yielding a sensitivity of 85.7% and a poor specificity of 24.8%. They considered that the accuracy of D-dimer test for the diagnosis of DVT was decreased by the administration of fondaparinux. Mitani et al. also drew a conclusion that D-dimer concentration alone had limited value as a hemostatic marker for early detection of DVT after TKA [35]. In their study, administration of fondaparinux was employed for 14 days postoperatively for thromboprophylaxis, and all the patients with DVT were in calf veins and asymptomatic. Our study comprised 200 patients undergoing TKA with rivaroxaban for thromboprophylaxis. The results showed an incidence of 14.5% DVT (29/200). All DVTs were in the leg veins and asymptomatic except one patient. Although D-dimer values were significantly higher in DVT patients at POD4 (*p* = 0.04), the cutoff value (3.8 mg/L) has a low sensitivity (58.6%), specificity (76%), positive predictive value (29.3%), and borderline negative predictive value (91.5%). To keep low false-positive numbers, the cutoff value of D-dimer must have a negative predictive value approaching 100% [2, 15]. We consider a negative predictive value of 91.5% is not high enough to effectively exclude DVT. Furthermore, our results showed a wide individual variation of D-dimer values on POD4 in DVT (+) group (1.66–9.81) as well as DVT (−) group (0.74–9.4). There were still some patients with positive venograms having low values of D-dimer. Therefore, the statistical difference in the D-dimer levels on POD4 might imply little clinical relevance. In this situation, there were no useful cutoff values to exclude DVT occurrence in these patients.

Another issue is the level of lower limb DVTs. Previous authors have demonstrated that plasma D-dimer test was useful to exclude proximal DVTs [36, 37]. Others reported its cost-effectiveness in the diagnosis of postoperative symptomatic DVTs [15]. On the opposite, the accuracy of D-dimer measurement for distal calf and asymptomatic thrombi was questionable [15, 33]. Therefore, D-dimers should be considered as a marker of larger intra- or extravascular fibrin formation after surgery [15]. Rivaroxaban has been shown to decrease the incidence of proximal DVTs after TKA [38]. Because of efficacy of oral anticoagulants, all 29 DVTs in our study were in the leg veins and asymptomatic except one. There were no proximal DVTs or pulmonary embolisms. The reduction of total thrombus volume and the amount of large emboli might result in poor accuracy of D-dimer tests in the prediction of postoperative DVT in the present study.

We recognized few limitations of this study. First, the number of the enrolled patients was small, and the lack of a control group limited our validation that rivaroxaban had an impact on postoperative D-dimer levels following TKA. In addition to being a highly selective and direct Factor Xa inhibitor, rivaroxaban was found to alter the fibrin network and increase porosities of the clot, resulting in a looser clot structure which became more susceptible to fibrinolysis [39]. These facts imply a more complex relationship between the anticoagulants and the hemostatic biomarkers. To the best of our knowledge, however, no study has focused on the influence of rivaroxaban on D-dimer test in the diagnosis of DVT before. Further investigations with larger sample size and a control group are needed. Second, this study was a retrospective review and no D-dimer test was conducted on the day when the ascending venography of both legs was performed. However, Bounameaux et al. has demonstrated that plasma D-dimer values did not differ between patients with or without DVT at the time of venography after TKA [33]. In that study, low-molecular-weight heparin was used for thromboprophylaxis. Third, most of the TKA patients followed a fast-track rehabilitation program and were discharged from the hospital averaged on POD4. While we noticed a significant correlation of D-dimer level on POD4 with the occurrence of DVT, we were unable to determine the longitudinal relationships between D-dimer concentrations with the development of DVT in the following course after discharge. That being said, the study still has its strengths. It is the first report focusing on the diagnostic efficacy of D-dimer test on DVT in TKA patients treated with rivaroxaban. All the patients in the study completed a 2-week chemical prophylaxis without undesirable complications. In addition, all the DVTs were documented by ascending venography.

## Conclusions

Our study showed that rivaroxaban was effective on reducing DVT in patients undergoing TKA. Because all the DVTs occurred in the leg veins, decreased thrombus volume and size might result in poor accuracy of plasma D-dimer test in prediction or diagnosis of postoperative DVT.

## Abbreviations

ASA: American Society of Anesthesiologists
AUC: Area under curve
BMI: Body mass index
DVT: Deep vein thrombosis
NPV: Negative predictive value
POD: Postoperative day
PPV: Positive predictive value
ROC: Receiver Operating Characteristics
TKA: Total knee arthroplasty
VTE: Venous thromboembolism
